# Suppressing material loss in the visible and near-infrared range for functional nanophotonics using bandgap engineering

**DOI:** 10.1038/s41467-020-18793-y

**Published:** 2020-10-07

**Authors:** Mingsong Wang, Alex Krasnok, Sergey Lepeshov, Guangwei Hu, Taizhi Jiang, Jie Fang, Brian A. Korgel, Andrea Alù, Yuebing Zheng

**Affiliations:** 1 Walker Department of Mechanical Engineering and Texas Materials Institute, The University of Texas at Austin, Austin, TX 78712 USA; 2 Photonics Initiative, Advanced Science Research Center, City University of New York, New York, NY 10031 USA; 3 Department of Physics and Engineering, ITMO University, St. Petersburg, 197101 Russia; 4 Department of Electrical and Computer Engineering, National University of Singapore, Singapore, 117583 Singapore; 5 McKetta Department of Chemical Engineering, The University of Texas at Austin, Austin, TX 78712 USA; 6 Physics Program, Graduate Center, City University of New York, New York, NY 10016 USA

**Keywords:** Nanophotonics and plasmonics, Nanoparticles, Nanoparticles

## Abstract

All-dielectric nanostructures have recently opened exciting opportunities for functional nanophotonics, owing to their strong optical resonances along with low material loss in the near-infrared range. Pushing these concepts to the visible range is hindered by their larger absorption coefficient, thus encouraging the search for alternative dielectrics for nanophotonics. Here, we employ bandgap engineering to synthesize hydrogenated amorphous Si nanoparticles (a-Si:H NPs) offering ideal features for functional nanophotonics. We observe significant material loss suppression in a-Si:H NPs in the visible range caused by hydrogenation-induced bandgap renormalization, producing strong higher-order resonant modes in single NPs with *Q* factors up to ~100 in the visible and near-IR range. We also realize highly tunable all-dielectric meta-atoms by coupling a-Si:H NPs to photochromic spiropyran molecules. ~70% reversible all-optical tuning of light scattering at the higher-order resonant mode under a low incident light intensity is demonstrated. Our results promote the development of high-efficiency visible nanophotonic devices.

## Abstract (web summary)

Large absorption of high-index semiconductors has hindered the application of all dielectric nanostructures in the visible range. Here, the authors present bandgap-engineered hydrogenated amorphous Si nanoparticles with Q-factors up to 100 and their integration with photochromic molecules as tunable meta-atoms.

## Introduction

Light trapping is crucial in a plethora of photonic applications, including lasers^1^, sensing^2^, and harmonic generation^3^, to name just a few. High-quality resonators facilitate light trapping in the form of localized resonant modes for a time ~2*Q*/*ω*_0_, where *Q* is the quality factor of the mode and *ω*_0_ is its eigenfrequency. In practice, the *Q* factor of a single standing resonance can be defined via its resonance linewidth at half maximum (Δ*ω*) as *Q* = *ω*_0_/Δ*ω*, implying that a higher *Q* factor possesses narrower resonant lines. Optical resonators supporting high-*Q* modes include microdisk resonators^4–8^, microspheres^9^, Bragg reflector microcavities^10^, and photonic crystals^11–13^, whose high *Q* factors can go up to ∼10^3^–10^6^. However, these microscale dielectric resonators are bulky and exhibit modest light–matter interactions when averaged over their large sizes.

Functional nanophotonics requires light localization and hence high *Q* factors in subwavelength optical resonators. To this end, nanoscale light trapping based on plasmonic resonances of metal nanoparticles^14–21^ and on Mie resonances of high-index dielectric nanostructures^2,22^ has been explored. However, the *Q* factor of these nanoresonators is limited by radiative and material losses, and it does not exceed a few tens in the visible range. Although radiative losses can be suppressed with the tailoring of weakly scattering states, such as bound states in the continuum (BIC)^23–26^, the material (dissipation) loss requires a fundamentally different approach. High-index dielectric resonant NPs look especially promising due to their lower material loss compared to plasmonic nanoparticles^14–19,27–30^, enabling low-loss photonic devices, e.g., nanoantennas, metalenses, and lasers^22,31–37^. Recent studies have also revealed the importance of high-index dielectric nanostructures as versatile platforms for active nanophotonics^38–43^. In contrast to plasmonic structures, where tailoring of the magnetic optical response is a challenging task, the response of high-index dielectric NPs is governed by both magnetic and electric resonances in visible and NIR range^22,44^. Among high-index dielectrics, Si-based NPs have been of particular interest for nanophotonic applications^31^, because Si is the choice material for the established semiconductor industry, where fully developed processing and characterization tools can readily be applied to create future CMOS-compatible integrated photonic and electronic systems based on a single-material platform^31,44,45^.

Theoretical studies have predicted enhanced *Q* factors for high-order multipole modes in low-loss high-index dielectric NPs compared with the fundamental dipole mode, because of reduced radiation loss^22,45–47^. However, such modes are fragile and very sensitive to material losses, hence they are hardly observable in practice. For example, crystalline silicon (c-Si) has a broad optical absorption in the wavelength range from 300 to 1150 nm, with an absorption coefficient larger than 1 × 10^3^ cm^−1^ over the whole visible region^48^ that reduces *Q* factor of the fundamental magnetic dipole (MD)^31^ and suppresses all high-order modes. This obstacle has prevented the development of low-loss and compact photonic devices for the visible region, and prompts a further search for alternative materials.

Recent studies have revealed the power of bandgap engineering for tailoring materials with superb optical properties^49^. For example, in ref. ^50^, it has been reported that increasing the atom spacing may significantly reduce material loss in metal nanoparticles. Another example is the optical properties of phase change materials, in which the amorphous phase exhibits lower material losses^51^. Other studies suggest that the optical losses of materials can be reduced via the formation of a forbidden phonon gap induced by random phononic structures^52^. In this paper, we employ bandgap engineering to tailor hydrogenated amorphous Si nanoparticles (i.e., a-Si:H NPs) and endow them with low dissipation in the visible range. First, we controllably vary the hydrogen concentration and the crystalline structure from crystal to amorphous and demonstrate that, similar to the amorphous phase of phase change materials, a-Si:H NPs exhibit smaller material loss. Next, based on single-nanoparticle spectroscopy of a-Si:H NPs, we report the experimental observation of strong magnetic quadrupole (MQ) and magnetic octupole (MO), with dominant scattering spectral peaks in the visible and NIR regions in subwavelength nanoparticles. In recent years, a-Si:H has been explored as a material platform for nanophotonic structures, including waveguides and nanocavities^37,53–55^. However, these studies mainly focused on the NIR and IR regions. Although a few more recent studies reported a-Si:H nanogratings and nanodisks for visible-wavelength-range applications, reduced loss in a-Si:H has not been fully appreciated and tailored to the visible region, and no higher-order Mie resonances have been explored or observed^56,57^.

Here, we show that MO scattering modes have *Q* factors up to 100 along with prominent peaks in the scattering spectra, which so far have been challenging to realize in experimental nanophotonics. Given the enhanced light–matter interactions, we are able to observe high tunability of the supported resonances by simply adjusting the hydrogen concentration. It should be mentioned that some studies have reported the observation of high-order modes in Si nanostructures in the visible region^44,58^. However, the spectral intensities arising from high-order modes in these studies are weak and their *Q* factors do not exceed a few tens, owing to aforementioned reasons. Finally, in order to demonstrate the unique opportunities offered by our approach to low-loss functional nanophotonics, we show that the coupling of strong higher-order scattering resonances to a single a-Si:H meta-atom with spiropyran (SP) photochromic molecules results in switchable ~70% tuning of the scattering peak intensity upon changing the state of the photochromic molecules between transparent SP and colored merocyanine (MC) states. This significant all-optical tunability is achieved using a non-laser light source with low incident light intensity.

## Results

### Bandgap engineering of low-loss hydrogenated a-Si nanoparticles

**Fig. 1:**
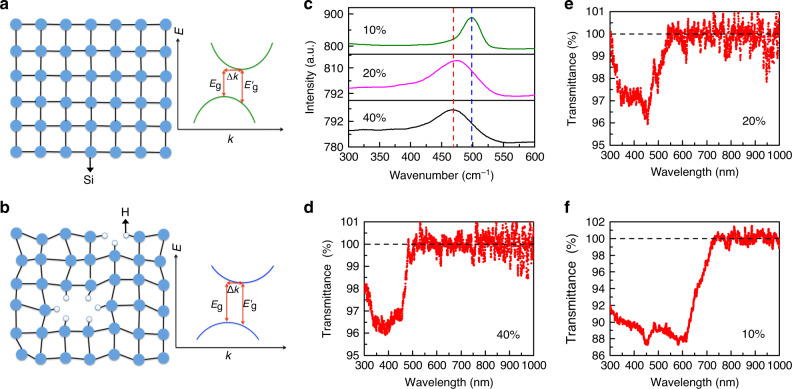
Bandgap engineering of low-loss hydrogenated a-Si nanoparticles. **a**, **b** Two-dimensional schematic representations of the atomic arrangement and the bandgap of c-Si (**a**) and a-Si:H (**b**). *E* is the energy, and *k* is the wave vector. *E*_g_ is the indirect bandgap, *E*_g_′ is the direct transition, and Δ*k* is the wave-vector mismatch. **c** Raman spectra of a-Si:H NPs with 40, 20, and 10% of hydrogen. The blue dashed line indicates the peak position of 495 cm^−1^, and the red dashed line labels the peak position of 470 cm^−1^. **d**–**f** Transmission spectra of a-Si:H NP(40) (**d**), a-Si:H NP(20) (**e**), and a-Si:H NP(10) (**f**) in ethanol after removing scattering signals.

It is known that the bandgap in Si is induced from short-range ordered structures of neighboring Si atoms, which lead to *sp*^3^-hybridization of s and p valence electrons, forming two bands with decreasing interatomic distance and an indirect bandgap. The bandgap energy of a-Si:H, therefore, depends on the interaction between the *sp*^3^ hybrids of neighboring Si atoms (Fig. 1a), and it is sensitive to the interatomic distance of Si–Si bonds^59,60^. As schematically shown in Fig. 1b, the voids in a-Si:H brought by hydrogen atoms cause a distortion of Si–Si bond, which leads to different bonding angles, smaller Si–Si distances and thus a larger bandgap, compared to c-Si. c-Si has an indirect bandgap (*E*_g_) of 1.16 eV (which corresponds to the wavelength in free space of 1069 nm) and a forbidden direct transition (*E*_g_′) of 3.2 eV (wavelength is 387 nm)^48^. As we demonstrate below, by tuning the hydrogen concentration, a-Si:H NPs can obtain the absorption bandgap larger than 1.77 eV (700 nm) via bandgap engineering, and thus sustain a lower loss and strong higher-order scattering modes with high *Q* factors in the visible range.

The a-Si:H NPs studied here were fabricated by chemical synthesis (detailed information is in “Methods” section)^59,61^. The SEM images of a-Si:H NPs are shown in Supplementary Figs. 1–3. Raman spectroscopy was employed to verify that the distortion of the Si–Si bond, and the amorphous nature of a-Si:H NPs increases with an increase of hydrogen concentration. As shown in Fig. 1c, when the hydrogen concentration is 10 at.%, the Raman peak is at ~495 cm^−1^ and it is relatively narrow (see the green spectrum in Fig. 1c). This peak is brought by the appearance of 1–2 nm nanocrystalline Si domains in the amorphous matrix^59^. As the hydrogen concentration increases, the Raman peak becomes broader and shifts to ~470 cm^−1^, which is associated with a transverse optical phonon mode of a-Si and stretching vibrational modes of Si–Si bonds in a silicon tetrahedron, thus indicating an increase in the disordered nature of a-Si:H NPs^59^.

The bandgap of a-Si:H NPs with different hydrogen concentrations is determined from measured transmission spectra of a-Si:H NPs, which are presented in Fig. 1d–f. The optical properties of a-Si:H film can be found in refs. ^60,62^ for comparison. Scattered signals are subtracted to obtain pure absorption signals (more detailed information is in Supplementary Note 2). Samples with 40 at.%, 20 at.%, and 10 at.% hydrogen (a-Si:H NP(40), a-Si:H NP(20), and a-Si:H NP(10)) have absorption bands starting at ~500, 560, and 710 nm, respectively. This confirms that the absorption bandgap of a-Si:H broadens as the hydrogen concentration decreases, and it can be tuned by varying hydrogen concentration. It should be mentioned that the transmission spectra are obtained through ensemble measurements, so each individual a-Si:H NP may have a slightly different absorption peak than those in Fig. 1d–f.

**Fig. 2:**
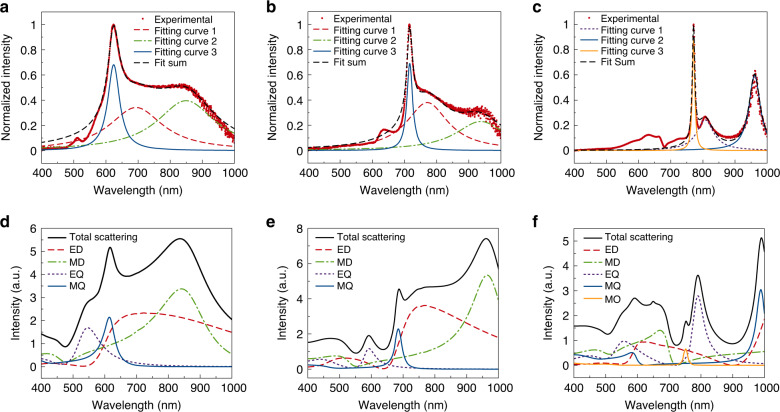
Single-nanoparticle optical spectroscopy. **a**–**c** Scattering spectra of a single a-Si:H NP(40) (radius of ~185 nm) (**a**), a-Si:H NP(20) (radius of ~160 nm) (**b**), and a-Si:H NP(10) (radius of ~207 nm) (**c**). The red dotted curve and black dashed curve are experimental data and fit summary, respectively. The red dashed curve, green doted-dashed curve, and blue curve are fitting curve 1, 2, and 3, respectively (**a**, **b**). Purple doted curve, blue curve, and dark yellow curve are fitting curve 1, 2, and 3, respectively (**c**). **d**–**f** Scattering spectra along with multipole decomposition calculated with Mie theory. The black curve, red dashed curve, green dashed-dotted curve, purple doted curve, and blue curve represent total, ED, MD, EQ, and MQ scattering, respectively (**d**–**f**). Dark yellow curve represents MO scattering (**f**).

Dark-field scattering spectra of single NPs were measured (see Supplementary Fig. 6 for the dark-field setup). Figure 2a–c shows the scattering spectra of a single a-Si:H NP(40), a-Si:H NP(20), and a-Si:H NP(10) of diameters 370, 320, and 414 nm, respectively. Three major peaks in each spectrum are observed, which were fitted into three Lorentzian peaks associated, as proven by our theoretical analysis presented below, with MQ resonances at 624 (Fig. 2a), 717 (Fig. 2b) and 961 nm (Fig. 2c), electric dipole (ED) resonances at 694 (Fig. 2a) and 771 nm (Fig. 2b), MD resonances at 849 (Fig. 2a) and 937 nm (Fig. 2b), a MO resonance at 771 nm (Fig. 2c), and an electric quadrupole (EQ) resonance at 807 nm (Fig. 2c), respectively. Remarkably, different from any previously reported SiNPs, MQ, and MO modes in our a-Si:H NPs show a large scattering cross section (SCS; see Fig. 2a–c, blue and dark yellow fitting curves). The strong MQ and MO scattering peaks are repeatable, and consistent with the prediction from Mie scattering theory^47^, confirming the low-dissipative nature of a-Si:H NPs in the visible and NIR range. It should be noted that the refractive index of a-Si:H NPs decreases with increased hydrogen concentration, due to the density reduction brought by hydrogenated nanovoids^60^. This causes a-Si:H NPs to have a smaller refractive index than a-SiNPs. The higher hydrogen concentration requires the size of a-Si:H NPs to be larger than the one of SiNPs to keep the Mie resonance peak positions aligned^22^. It is also worth noticing that new studies have been emerging focused on exploring moderate-refractive-index materials (*n* > 2) with low loss in the visible region, such as silicon-rich silicon nitride (SRN), titanium dioxide (TiO_2_), and gallium nitride (GaN), as platforms for high-efficient metasurfaces in the visible region^60,62–68^. Several SRN, TiO_2_, and GaN nanostructures have been fabricated through top-down approaches, such as SRN nanodisks^63,64^, TiO_2_ nanofins^65,66^, TiO_2_ nanoblocks^67,68^, and GaN nanopillars^69,70^. Distinct dipole Mie resonances were demonstrated in these nanostructures, but no higher-order modes were observed, possibly because of the limited quality of the nanostructures made via top-down approaches.

In addition, the hydrogen concentration modification here is entirely different from carrier doping, which can also effectively tune the optical response of high-index NPs^71^. Namely, unlike the Drude response of free-carrier doping, hydrogen voids induce a blueshift of the absorption band, which induces a significant decrease of the dissipative loss to shorter wavelengths. However, carrier doping increases the dissipative loss, which has been illustrated by a broadening of the resonance peaks and the formation of a metallic phase^71^.

Mie scattering theory is used to confirm the origin of each scattering peak and clarify how the Mie resonance peaks change, as a function of the hydrogen concentration. To model the permittivity of a-Si:H NPs, we use the Maxwell Garnett mixing formula

1 $$\varepsilon _{{\mathrm{a}} {\hbox{-}} {\mathrm{Si:H}}} = \varepsilon _{{\mathrm{a}} {\hbox{-}} {\mathrm{Si}}}\frac{{(\varepsilon _{{\mathrm{voids}}} + 2\varepsilon _{{\mathrm{a}} {\hbox{-}} {\mathrm{Si}}}) + 2f(\varepsilon _{{\mathrm{voids}}} - \varepsilon _{{\mathrm{a}} {\hbox{-}} {\mathrm{Si}}})}}{{(\varepsilon _{{\mathrm{voids}}} + 2\varepsilon _{{\mathrm{a}} {\hbox{-}} {\mathrm{Si}}}) - f(\varepsilon _{{\mathrm{voids}}} - \varepsilon _{{\mathrm{a}} {\hbox{-}} {\mathrm{Si}}})}},$$

where *f* is the volume fraction of hydrogenated voids and *ε*_voids_ denotes the permittivity of voids, which is assumed to be 1. We also slightly blueshift the permittivity of a-Si (shown in Supplementary Fig. 7) to model the hydrogenation. We have introduced this blueshift to take into account the bandgap renormalization caused by hydrogenation. By using *f* = 0.4 and a blueshift of 140 nm, and *f* = 0.65 and a blueshift of 90 nm, we obtain the best matching of simulated scattering peaks with experimental data of a-Si:H NP(40) (radius of ~185 nm), a-Si:H NP(20) (radius of ~160 nm), and a-Si:H NP(10) (radius of ~207 nm), respectively.

The optical response of fabricated spherical a-Si:H NPs with dielectric permittivity *ε*_a-Si:H_ and radius *R* are calculated by the Mie light scattering theory^72,73^, which gives the following expression for normalized SCS for particles made of a nonmagnetic material:

2 $$Q_{{\mathrm{sct}}} = \frac{2}{{(kR)^2}}\mathop {\sum}\limits_{l = 1}^\infty {(2l + 1)} \left( {|a_l|^2 + |b_l|^2} \right),$$

where *l* defines the order of resonant mode and *k* is the wavenumber in the surrounding material. For a single-component particle, the electric (*a*_*l*_) and magnetic (*b*_*l*_) scattering amplitudes are given by $a_l = R_l^{(a)}/\left( {R_l^{(a)} + iT_l^{(a)}} \right)$, $b_l = R_l^{(b)}/\left( {R_l^{(b)} + iT_l^{(b)}} \right)$, and functions *R*_*l*_ and *T*_*l*_ can be expressed through the Bessel and Neumann functions (see “Methods” for details). The simulated scattering spectra are shown in Fig. 2d–f. The *E*-field distributions at the scattering peaks at 616 nm in Fig. 2d, 687 nm in Fig. 2e, and 991 nm in Fig. 2f are shown in Supplementary Fig. 8a, b, d, respectively. The *E*-field distribution profiles reveal the MQ features at these peaks, while the *E*-field distribution profile at 769 nm shown in Supplementary Fig. 8c displays a MO feature.

We calculated the *Q* factor of the measured MQ, MO, and MD scattering peaks by the definition^31^ *Q* = *ω*_0_/Δ*ω*, discussed above. The calculated *Q* factor of MQ modes in Fig. 2a, b are 11 and 30, respectively. These *Q* factors are several times larger than those of MD modes (5 and 6 for Fig. 2a, b, respectively), suggesting that MQ modes can further boost light–matter interactions because of reduced radiation loss in a-Si:H NPs. The measured MQ scattering peaks are even more pronounced than those in simulations. The reason for the difference resides in the interplay of the resonant overlapping modes, resulting in complicated scattering power patterns. As a result, the amplitudes of the resonances in scattering measurements strongly depend on the experimental setup, whereas we focus on the total SCS in the simulations. Also, this difference between the simulated and measured amplitudes implies that the actual dissipative loss of a-Si:H NPs is smaller than the one obtained by blueshifting the *ε* dispersion of pure a-Si. Furthermore, in Fig. 2c, we observe the MO resonance with *Q* factor of ~100, which is ~10–20 times those obtained with plasmonic NPs at lower-order resonances (see Supplementary Note 5 for a detailed discussion).

It is worth noting that we address only material (dissipative) losses in our work. Hence, radiative losses represent the upper limit on the value of *Q* factor of dielectric nanoparticles. Radiative losses can be further reduced by utilizing, for example, quasi BIC, as recently demonstrated^74^. In order to further increase the *Q* factor of high-index nanostructures, approaches to suppress both types of losses should be applied simultaneously.

### Highly tunable hybrid meta-atom

To demonstrate the superiority of our results for low-loss functional nanophotonics, we show that the coupling of these strong higher-order scattering resonances in a single a-Si:H meta-atom with SP molecules facilitates actively tunable nanostructures possessing low tuning intensities.

In practice, high input powers are needed to achieve strong all-optical tunability in Si due to its weak optical tunability^75,76^. In addition, the interaction length in SiNPs is limited by their nanoscale size. Thus, to achieve sufficient optical tuning of SiNPs, the high incident light intensity is needed. For instance, the intensities used for optically tuning SiNPs via electron–hole plasma excitation and laser reshaping are ~10 GW cm^−2^ and ~21 MW cm^−2^, respectively^77–79^. High incident light intensities lead to considerable energy consumption, and can cause damage to SiNPs and surrounding materials. Therefore, all-optical tuning techniques relying on low intensities of incident light are desirable.

**Fig. 3:**
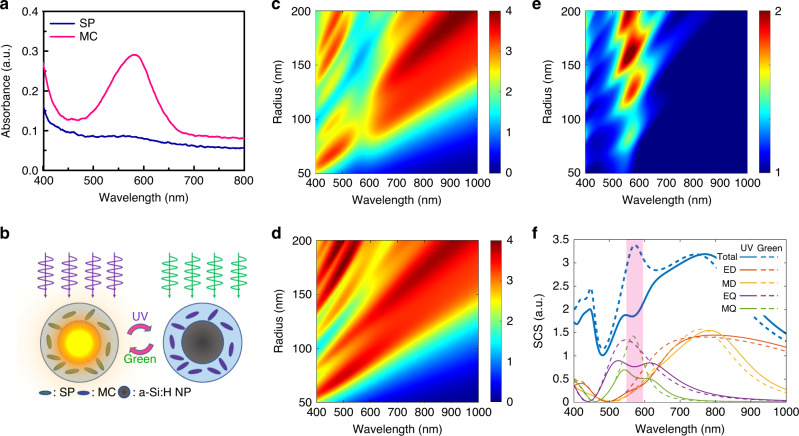
Designing of the highly tunable photochromic all-dielectric single-particle meta-atom. **a** Absorption spectra of molecules in SP (blue curve) and MC (pink curve) state. **b** Schematic representation of the meta-atom consisting of a-Si:H with 40% of hydrogen core and a shell of SP photochromic molecules. The meta-atom is designed to support a strong higher-order Mie resonance at the absorption resonance of the MC state. The photoisomerization from SP to MC state and from MC to SP state is caused by UV and green light excitation, respectively. **c**, **d** Scattering cross-section (normalized to geometric) map of a core–shell nanostructure (**c**) after UV modification, and (**d**) green light recovery as a function of wavelength and a-Si:H nanoparticle radius. **e** Ratio between scattering cross sections of the core–shell after green light recovery and UV modification. **f** Scattering cross section of the core–shell with the Si core radius of 130 nm and its multipole decomposition. Solid lines correspond to the core–shell after UV modification, dashed lines correspond to the core–shell after green light recovery. The thickness of the PMMA + SP shell is 100 nm.

Here, we demonstrate that coupling higher-order optical resonant modes of a-Si:H NPs with photoswitchable optical resonances of photochromic molecules^19,80,81^, such as SP molecules is a promising way to achieve low-intensity all-optical tuning in the visible range. The photochromism of SP is schematically displayed in Supplementary Fig. 11. The absorption spectra of molecules in SP form (blue curve) and MC form (pink curve) states are shown in Fig. 3a. Figure 3b schematically illustrates the investigating structure composed of the tailored a-Si:H NP, supporting strong MQ mode covered by SP molecules. The photoisomerization from SP to MC (from MC to SP) induced by UV (green) light excitation causes strong tuning of the scattering response of the single a-Si:H meta-atom from strong scattering to suppressed scattering, if the resonance of molecules in MC form is aligned with the MQ mode of the NP.

We start with the analytical optimization of geometry to achieve a pronounced effect of scattering tuning. To find the optimal layout, we fix the thickness of the shell (PMMA + SP) to 100 nm, and vary the core radius *R* from 50 to 200 nm. For analysis, we use the Mie theory for multilayered spheres^82^. The dielectric permittivity of the core is assumed to a-Si:H (40) find above, while the permittivity of PMMA + SP is obtained from experiments on transmission/reflection from the bare PMMA + SP film. The results of analytical calculations of SCS versus *R* and wavelength in the MC state are summarized in Fig. 3c. We observe a pronounced dip at 580 nm in the scattering spectrum caused by the interaction with the absorption peak of MC molecules. It should be noted that, although a-Si:H NP(10)s have the highest *Q* factor, their MQ modes exist in the far red side of the visible spectral range and NIR region, and are thus far away from the excitonic resonances of the photochromic molecules. Thus, in our studies we have chosen a-Si:H NP(40)s that can have strong MQ modes ~580 nm and better match with the resonances of the molecules, despite their smaller *Q* factors. In addition, lower *Q* factors don’t necessarily mean a weaker modulation in our system because a-Si:H NPs with lower *Q* factors interact with more photochromic molecules in the PMMA + SP film due to the weaker localization of the field.

As the form of the photochromic molecules changes, the scattering spectrum of the meta-atom is drastically transformed. In the fully SP form, the meta-atom possesses scattering peaks at 580 nm, Fig. 3d, caused by different resonant modes of the a-Si:H NP(40) at different radii. The ratio of SCS in both states presented in Fig. 3e gives the change of SCS. As expected, the coupling between MQ resonance and MC molecules demonstrates a more dramatic change than for the MD resonance. We see that a large tuning effect can be achieved when the MQ resonant mode of a meta-atom with a-Si:H NP(40) core radius of ~130 nm is at 580 nm. This drastic SCS variation is caused by the combination of the strong field enhancement of the MQ mode and the large dipole strength of the photochromic molecules in the SP state. Results of the mode decomposition of the structure in both states are presented in Fig. 3f.

**Fig. 4:**
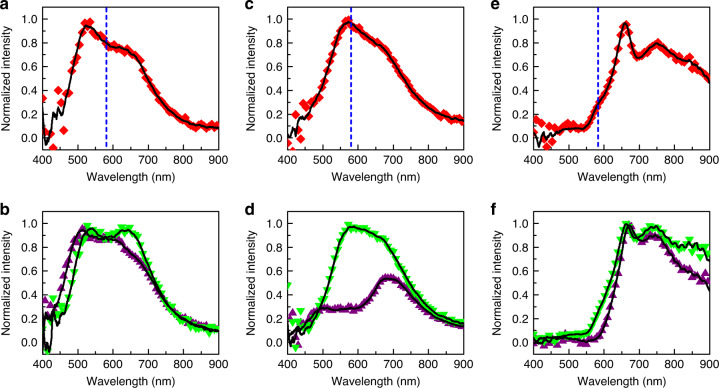
Tuning of the all-dielectric meta-atom. **a**–**f** Scattering spectra of two single a-Si:H NPs(40) (diameter: ~300 and ~325 nm, respectively) (**a**–**d**) and a single a-Si:H NP(20) (diameter: ~280 nm) (**e**, **f**) covered by PMMA + SP layer. **a**, **c**, **e** Right after the sample preparation. **b**, **d**, **f** After the exposure to UV light (purple triangle) and after the exposure to the green light (green triangle). The vertical dashed blue dashed line illustrates the absorption peak of MC molecules. The black curves are smoothed spectral curves.

To verify this enhanced tunability, the fabricated a-Si:H NPs have been covered by a spin-coated PMMA + SP film. Figure 4 shows the scattering spectra of two single a-Si:H NP(40)s and one single a-Si:H NP(20) covered by PMMA + SP film. All scattering spectra have been normalized by dividing the transmission spectra of PMMA + SP film without a-Si:H NPs under the same power of dark-field light source to eliminate the contribution of bare molecular absorption. Before light irradiation, these three NPs in PMMA + SP film have MQ peaks centering at 522, 573, and 660 nm, respectively. The MQ peaks of the middle one match with the molecular absorption at 580 nm, while the other two have a large detuning from it. A fluorescence illuminator (mercury lamp) with bandpass filters was used to generate UV and green light, in order to switch the molecules from the SP state to the MC state and vice versa. The estimated maximum light intensities employed in the experiment are 3.8 and 1.1 × 10^2^ W cm^−2^ for UV and green light, respectively (detailed information in “Methods”). These intensities are significantly lower than those used in reported studies of all-optical tuning of SiNPs. For instance, the intensities used for optically tuning SiNPs via electron–hole plasma excitation and laser reshaping are ~10 GW cm^−2^ and ~21 MW cm^−2^, respectively^77,78^.

We observe a remarkable tuning (~70%) of scattered power in the matched scenario near the MQ (Fig. 4c) scattering peak in Fig. 4d. The observed tunability is reversible and is caused by the large field enhancement of the MQ resonant mode. However, when there is a detuning (Fig. 4a, e), negligible scattering change occurs when SP molecules are switched to MC state, i.e., after UV light exposure for ~1.5 min (Fig. 4b, f). This change vanishes when the molecules are switched back to SP form by irradiating the sample with the green light for ~3 min, as shown by Fig. 4b, d, f. This photoswitchable variation confirms that the strong modulation of MQ scattering observed here is derived from the interaction between MQ mode and photochromic molecules. The switching speed of SP molecules in the PMMA film is relatively slow under a low incident light intensity. However, the ultrafast photoswitching of SP molecules has been demonstrated via femtosecond laser, and the entire cycle takes <40 ps (refs. ^83,84^). Thus, we think that fast photoswitchable modulation of the optical response of the suggested hybrid meta-atom can be achieved by modifying the environment around SP molecules, and using advanced excitation sources. It is also worth noting that in many vital applications the ultrafast response is not necessarily required, but the strong tunability and reconfigurability are practically needed. The application examples include reconfigurable metasurfaces and metalenses, optical encryption, anti-counterfeiting, smart windows, and biomedical sensing.

Reversible modulation of the scattering intensity was demonstrated in our system, as shown in Supplementary Fig. 12. The result indicates that no sign of photodegradation was observed after five cycles of photoswitching. We should mention that our samples are not optimized for practical applications requiring highly robust performance. Many techniques that strongly improve the robustness of isomerization cycles, such as the covalent attachment of SP molecules to inorganic or macromolecular carriers, have been demonstrated^80^. We believe that more stable hybrid meta-atoms can be realized by applying those techniques.

## Discussion

In summary, we have used bandgap engineering to tailor a-Si:H NPs with low dissipation losses in the visible range. As a result, strong higher-order magnetic multipole resonances in the visible and NIR ranges with *Q* factors up to 100 have been experimentally demonstrated in tailored a-Si:H NPs. The appearance of strong magnetic multipole scattering modes stems from the larger bandgap of a-Si:H NPs compared to pure SiNPs. The bandgap of a-Si:H NPs can be tuned by changing the hydrogen concentration. Strong magnetic multipole scattering modes with narrow linewidths in the visible and NIR regions open new opportunities to use nanostructures made of low-loss high-index materials for light manipulation, exploring light–matter interactions, and tunable optical devices. We show that coupling such strong higher-order scattering resonances in a single a-Si:H meta-atom with photochromic SP molecules results in ~70% tuning of the scattering peak intensity upon switching the photochromic molecules between transparent SP and colored MC states. This photochromatic all-optical tuning of SiNPs requires drastically lower incident light intensity than other reported methods. Our results pave the way to novel devices based on highly tunable all-dielectric single nanostructures with low-intensity requirements.

## Methods

### Sample preparation

A 10 mL titanium batch reactor (High-Pressure Equipment Company (HiP Co.)) was used for the synthesis. First, 21 µL trisilane (Si_3_H_8_, 100%, Voltaix) and *n*-hexane (anhydrous, 95%, Sigma-Aldrich) were loaded in the reactor in a nitrogen-filled glove box. The amount of *n*-hexane loaded in the reactor is associated with the reaction pressure inside the reactor during the heating process. In all reactions, the pressure was kept at 34.5 MPa (5000 p.s.i.). The different hydrogen concentration in these a-Si:H NPs is determined by different reaction temperatures^59^. For example, the a-Si:H nanoparticles with a hydrogen concentration of 40% were synthesized at a temperature of 380 °C (ref. ^59^). After adding the reagents, the reactor was sealed by using a wrench inside the glove box. Then a vice was used to tightly seal the reactor after removing it from the glove box. The reactor was heated to the target temperature in a heating block for 10 min to allow the complete decomposition of trisilane. After the reaction, an ice bath was used to cool the reactor to room temperature. Colloidal a-Si:H NPs were then extracted from the opened reactor. The nanoparticles were washed by chloroform (99.9%, Sigma-Aldrich) using a centrifuge (at 8000 r.p.m. for 5 min).

There are three main reasons why we didn’t study a-Si:H NPs with a hydrogen concentration <10%: (1) a minimum amount of hydrogen is needed to achieve a large enough bandgap change to dramatically reduce the absorption loss of a-Si:H in the visible region; (2) 10% is the smallest hydrogen concentration for the bottom-up approach we employed to fabricate a-Si:H nanospheres; and (3) it is hard to precisely measure the hydrogen concentration of <10% with thermogravimetric analysis.

The SP + PMMA film is prepared by mixing SP (Sigma-Aldrich) molecules with PMMA (Sigma-Aldrich) with a weight ratio of 1:1 in chlorobenzene (2 wt % of SP in chlorobenzene). Then a spin coater (Laurell) is used to coat the mixture on a-Si:H NPs at 2000 r.p.m. for 1 min.

### Optical measurements

Before the scattering measurement, a-Si:H NPs were drop coasted on a bare glass substrate. An inverted microscope (Ti-E, Nikon) with a spectrograph (Andor), an EMCCD (Andor) and a halogen white light source (12 V, 100 W) was employed to measure the scattering spectra of single a-Si:H NPs on the bare glass substrate^18,19^. Raman spectra of a-Si:H NPs were measured by the Witec Micro-Raman Spectrometer. A UV-VIS-NIR spectrometer (Ocean Optics) was used to measure the transmission of a-Si:H NPs in ethanol. An epi-fluorescence illuminator (mercury lamp, Nikon) and bandpass filters (central wavelength: 350 nm (UV) and 540 nm (green), bandwidth: 50 nm (UV) or 25 nm (green)) are used to generated UV and green light. The maximum power of UV and green light was measured by a power meter (Thorlabs). Since the mercury lamp is an incoherent light source and the incident light covers the whole rear aperture of the objective, the beam spot diameter used to calculate the incident light intensity is obtained by: *d*_spot_ = *d*_aperture_/magnification, where *d*_aperture_ is the diameter of the objective rear aperture and equal to 6.5 mm and magnification is 100 times.

### Analytical and numerical simulations

The optical response of a Si nanoparticle with dielectric permittivity *ε* = *n*^2^ (*n* is the refractive index of the nanoparticle material) and a radius *R* located in the free space can be treated via Mie light scattering theory^72,73^, which gives the following expression for normalized scattering [$Q_{{\mathrm{sct}}} = P_{{\mathrm{sct}}}/(\pi R^2I)$] cross section for particles made of a nonmagnetic material:

3 $$Q_{{\mathrm{sct}}} = \frac{2}{{(kR)^2}}\mathop {\sum}\limits_{l = 1}^\infty {(2l + 1)} \left( {|a_l|^2 + |b_l|^2} \right),$$

where *l* defines the order of partial wave, *k* is the wavenumber *k* = *ωn*_m_/*c*, and $\varepsilon _{\mathrm{m}} = n_{\mathrm{m}}^2$ is the dielectric permittivity of the surrounding medium. The quantity *P*_sct_ denotes the scattering power, *I* is the excitation intensity, *c* is the speed of light, and *ε*_0_ is the dielectric constant.

For a single-component particle, the electric and magnetic scattering amplitudes are given by

4 $$a_l = \frac{{R_l^{(a)}}}{{R_l^{(a)} + iT_l^{(a)}}},\quad b_l = \frac{{R_l^{(b)}}}{{R_l^{(b)} + iT_l^{(b)}}},$$

and functions *R*_*l*_ and *T*_*l*_ can be expressed in the following form:

5 $$R_l^{(a)} = n\psi _l^\prime (kR)\psi _l(nkR) - \psi _l(kR)\psi _l^\prime (nkR),$$

6 $$T_l^{(a)} = n\chi _l^\prime (kR)\psi _l(nkR) - \chi _l(kR)\psi _l^\prime (nkR),$$

7 $$R_l^{(b)} = n\psi _l^\prime (nkR)\psi _l(kR) - \psi _l(nkR)\psi _l^\prime (kR),$$

8 $$T_l^{(b)} = n\chi _l(kR)\psi _l^\prime (nkR) - \chi _l^\prime (kR)\psi _l(nkR).$$

Here, $$\psi _l(x) = \sqrt {\frac{{\pi x}}{2}} J_{l + 1/2}(x),$$$$\chi _l(x) = \sqrt {\frac{{\pi x}}{2}} N_{l + 1/2}(x),$$$$J_{l + 1/2}(x)$$, and $$N_{l + 1/2}(x)$$ are the Bessel and Neumann functions, and prime means derivation.

The Mie scattering coefficients can be expressed similarly and can be found in refs. ^72,85^.

The optical properties of the nanostructures in the optical frequency range have been studied numerically by using CST Microwave Studio. CST Microwave Studio is a full-wave 3D electromagnetic field solver based on a finite-integral time-domain solution technique. A nonuniform mesh was used to improve accuracy in the vicinity of the Si nanoparticles, where the field concentration was significantly large.

## Supplementary information

Supplementary Information

## Data Availability

The data that support the findings of this study are available from the corresponding authors upon reasonable request.
