# Supplementary material for: Suppressing material loss in the visible and near-infrared range for functional nanophotonics using bandgap engineering
Source: Nat Commun. 2020 Oct 7;11:5055. doi: 10.1038/s41467-020-18793-y (PMC7542432; doi:10.1038/s41467-020-18793-y)
Supplement: Supplementary file 1 — Supplementary Information [file 41467_2020_18793_MOESM1_ESM.pdf]

Supplementary Information

Suppressing material loss in the visible and near-infrared range for functional nanophotonics using bandgap engineering

Wang et al.

Supplementary Note 1. SEM images of a-Si:H NPs

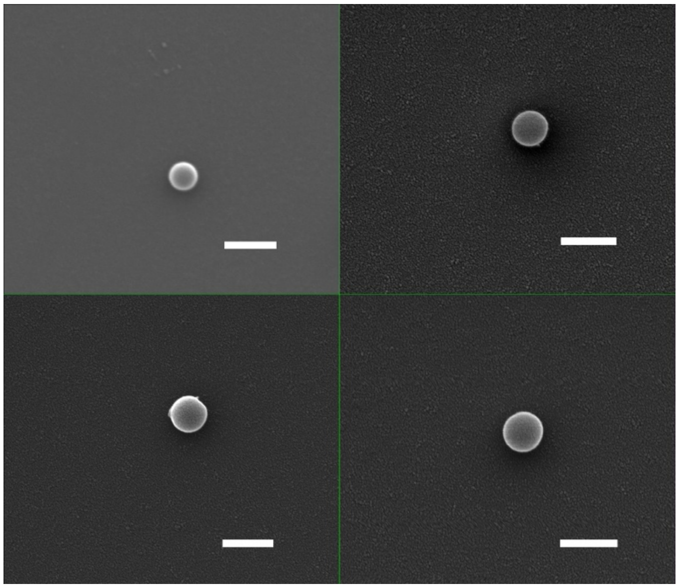

Supplementary Figure 1| SEM images of a-Si:H NPs with 40% hydrogen (a-Si:H(40)). Scale bar is 500 nm.

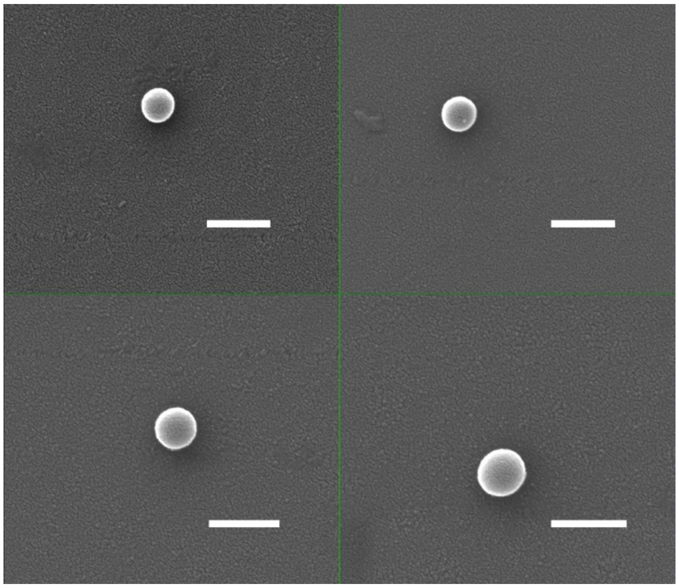

Supplementary Figure 2| SEM images of a-Si:H NPs with 20% hydrogen (a-Si:H(20)). Scale bar is 500 nm.

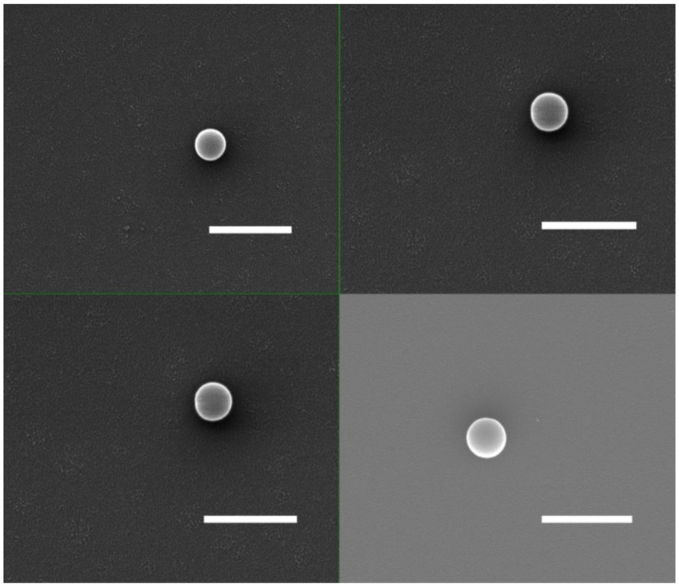

Supplementary Figure 3│ SEM images of a-Si:H NPs with 10% hydrogen (a-Si:H(10)). Scale bar is 1 μm.

Supplementary Note 2. Transmission spectra of a-Si:H NPs

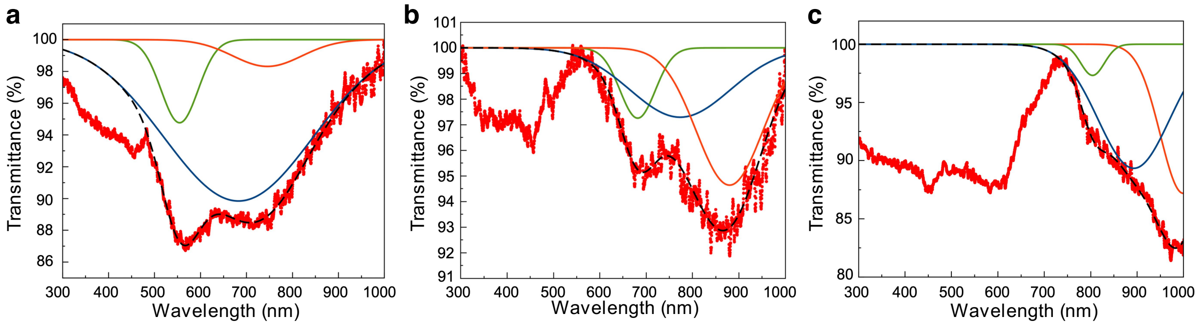

Supplementary Figure 4│ a,b,c, Transmission spectra of a-Si:H NPs with 40% (a), 20% (b) and 10% (c) hydrogen in ethanol. Green curves, red curves, blue curves are fitting Gaussian curves for magnetic quadrupole mode (MQ), electric dielectric dipole mode (ED), and magnetic dipole mode (MD), respectively. Black dashed curves are fit summary. The measured size ranges (i.e., NP diameters) are from~261 nm to ~370 nm, from ~258 nm to ~320 nm, and from ~356 nm to ~438nm for (a), (b), and (c), respectively.

a-Si:H NPs can strongly scatter light due to the excitation of Mie resonances[1,2], so transmission spectra show the optical extinction possessing both absorption and scattering signals. Supplementary Figure 4 shows that transmission spectra of a-Si:H NPs have several dips, those dips at longer wavelength are brought by optical scatterings and can be fitted into three Gaussian peaks. It should be mentioned that Gaussian distribution instead of Lorentzian distribution being used for fitting transmission dips is because transmission spectra were obtained from multiple particles in the solvent. Fitting dip positions in Supplementary Figure 4 match well with corresponding magnetic quadrupole (MQ), electric dipole (ED) and magnetic dipole (MD), magnetic octupole (MO), and electric quadrupole (EQ) scattering peak positions of a-Si:H NPs (40, 20 and 10), as shown in Fig. 2. The bare optical absorption of a-Si:H NPs can be gained by removing those scattering signals. It should also be noted that the refractive index of ethanol is about 1.4 and adding ethanol can shift the peak positions of dipole modes to slightly longer wavelengths compared to the ones in air[3]. But this does not affect the bandgap of a-Si:H and thus does not shift the absorption peak derived from the material loss.

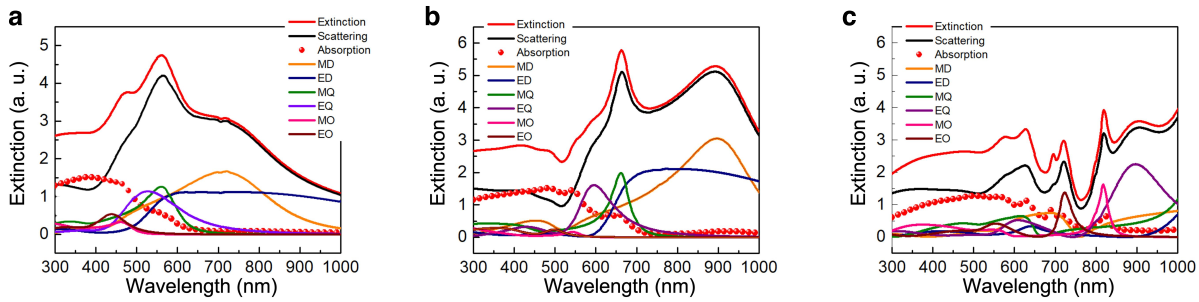

Supplementary Figure 5│ a,**b**,**c**, Simulated extinction spectra of a-Si:H NPs(40) (diameters: 300 nm) (**a**), a-Si:H NPs (20) (280 nm) (**b**), and a-Si:H NPs(10) (diameters: 420 nm) (**c**) in the solvent. EO represents the electric octupole.

In order to further verify the optical absorption of a-Si:H NPs, we simulated the extinction spectra of a-Si:H NPs with different hydrogen concentrations through Mie theory and used the same method mentioned in the main text to obtain their permittivity and dispersion. Since the transmission spectra were obtained through ensemble measurements, we chose a-Si:H NPs (40, 20 and 10) having three representative sizes (diameters: 300nm, 280nm and 420 nm) to calculate extinction spectra, respectively. The obtained extinction spectra with absorption components and absorption components are shown in Supplementary Figure 5. The simulated spectra are in good agreement with experimental results.

Supplementary Note 3. Optical setup

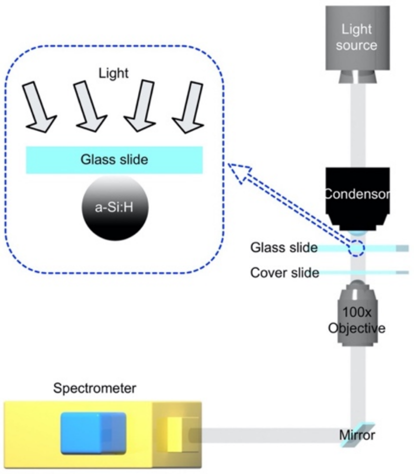

Supplementary Figure 6| Schematics of the optical setup and sample configuration for the single-nanoparticle dark-field scattering spectroscopy. The items in the schematics are not drawn to scale.

Supplementary Note 4. Permittivity dispersion of a-Si:H (40) and E-field distribution

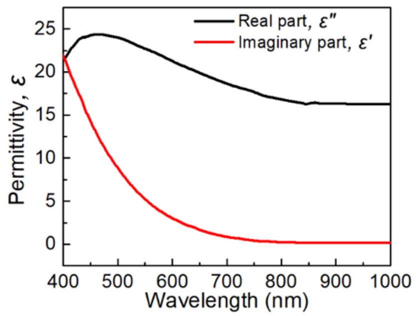

Supplementary Figure 7| Dependence of permittivity dispersion (ε) of a-Si:H (40) on the wavelength. Permittivity contains the real part (ε') and imaginary part (ε''): $\varepsilon = \varepsilon' + i\varepsilon''$. The permittivity is calculated through Eq.(1) from the main text with the experimental data taken into account.

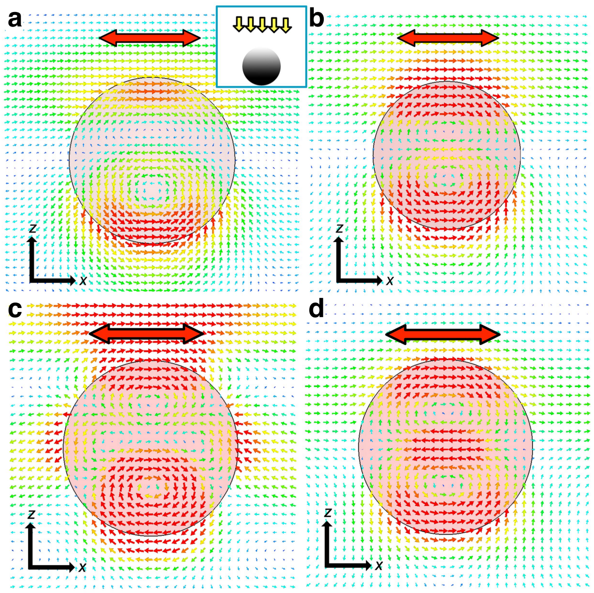

Supplementary Figure 8| **a**,**b**, Numerically calculated E-field distribution profiles at 616 nm (**a**) in Fig. 2d and at 687 nm (**b**) in Fig. 2e. **c**,**d**, Numerically calculated E-field distribution profiles at 769 nm (**c**) and at 991 nm (**d**) in Fig. 2f. The incident light (yellow arrows) irradiates on a-Si:H

NPs from the top as shown by the inset, and the red arrow indicates the light polarization. The E-field distributions are normalized equivalently so that the red color corresponds to the maximal value of 5.

Supplementary Note 5. Demonstration of MQ and MO modes

Scattering spectra of a-Si:H (40 and 20) NPs with different diameters are shown in Supplementary Figure 9. These results show that the MQ scattering generally diminishes when the scattering peak blueshifts towards the absorption band of a-Si:H NPs, where dissipative loss and the imaginary part of permittivity (ε'') increase significantly. It is also observed that the MQ scattering fades faster than the MD scattering, as expected because MQ modes have larger stored energy, and therefore they are more prone to loss. This explains the lack of observation of these modes in previous experiments using conventional materials. In order to statistically reveal this trend, we show the dependence of the ratio between MQ and MD scattering peak intensity versus wavelength in Supplementary Figures 9c and d. A steady decrease of MQ vs MD ratio is observed when the wavelength shifts towards the absorption peak of a-Si:H NPs.

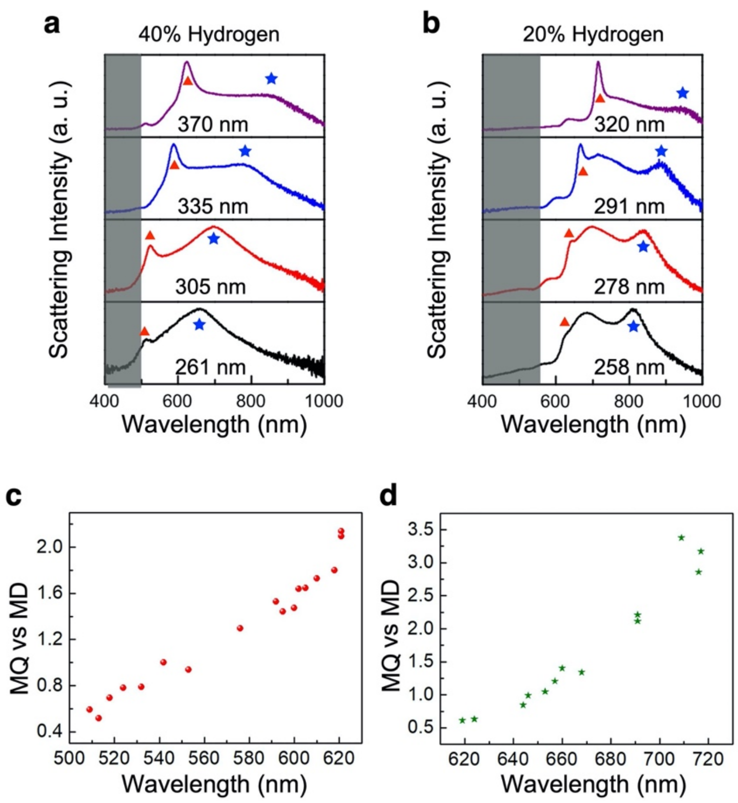

Supplementary Figure 9│ a, Scattering spectra of single a-Si:H(40) NPs. Diameters of NPs are 261 nm (black curve), 305 nm (red curve), 335 nm (blue curve) and 370 nm (purple curve). **b**, Scattering spectra of single a-Si:H(20) NPs. Diameters of NPs are 258 nm (black curve), 278 nm (red curve), 291 nm (blue curve) and 320 nm (purple curve). The red triangles and blue stars label the peak positions of MQ and MD scattering, respectively. **c**,**d**, Dependences of the ratio between MQ and MD scattering peak intensity on the peak position of MQ for a-Si:H(40) NPs (**c**) and a-Si:H(20) NPs (**d**).

We can also draw the same conclusion by comparing a-Si:H NPs with different hydrogen concentrations. Both the blue curve in Supplementary Figure 9a and the back curve in Supplementary Figure 9b show a MQ resonance at around 600 nm and a MD resonance at about 800 nm, while the MQ vs MD ratio of a-Si:H(40) NPs is larger than the one of a-Si:H(20), because the absorption of a-Si:H(40) NPs is at a shorter wavelength. As an additional remarkable knob, these results indicate that the minimum wavelength of the MQ scattering peak can be tuned by varying the hydrogen concentration of a-Si:H NPs.

By further comparing Supplementary Figure 9a with Supplementary Figure 9b, it is observed that both MQ and MD scattering peaks are narrower when the hydrogen concentration is lower. The linewidth of the scattering peaks follows[4]:

$$\gamma_\ell = \frac{q^{2\ell+1}(\ell+1)}{[\ell(2\ell-1)!!]^2 (d\epsilon/d\omega)_\ell} \tag{1}$$

where q is a size parameter and equates $2\pi R/\lambda$, ℓ is the order of the optical mode, and ω is frequency. $\epsilon = \epsilon_\mathrm{p}(\omega)/\epsilon_\mathrm{m}(\omega)$, where $\epsilon_\mathrm{p}(\omega)$ and $\epsilon_\mathrm{m}(\omega)$ is the permittivity of the particle and the medium, respectively. This equation reveals that the value of γ_ℓ decreases with a decrease in q and an increase in ℓ, assuming that ϵ and ω are fixed. When n is larger than 2[1,5], $2R$ is approximately proportional to λ/n at Mie resonance. Therefore, q is inversely proportional to n at the MQ resonances; i.e., γ_ℓ decreases when n becomes larger. Since the decrease in hydrogen concentration leads to a larger refractive index, a-Si:H NPs with lower hydrogen concentration have narrower MQ scatterings and higher Q-factors.

Higher-order multipole scattering modes, such as the magnetic octupole (MO) scattering, were also observed in the NIR region by further reducing the hydrogen concentration and enlarging the particle size. Fig. 2c shows scattering spectra of an a-Si:H(10) NP with a diameter of ~414 nm, the major three peaks can be again fitted with Lorentzian models. Simulation results (particle diameter: 400 nm, f=0.7 and a 50 nm blueshift of ε dispersion) in Fig. 2f reveal that the three peaks at 769 nm, 801 nm and 991 nm are MO, EQ and MQ, respectively. Scattering spectra of a-Si:H(10) NPs with diameter from 356 nm to 438 nm are shown in Supplementary Figure 10a. These spectra highlight that MO scattering is stronger than MQ scattering when it is away from the absorption peak, and MO gradually decreases when the peaks shift towards the absorption peak, as illustrated by the dependence of MO vs MQ ratio on the wavelength for a-Si:H(10) in Supplementary Figure 10b. We calculated the Q-factors of the MO scattering peaks in Supplementary Figure 10a, and obtained the highest Q-factor of 100, which is ~10-20 times of those obtained with plasmonic NPs at lower-order resonances.

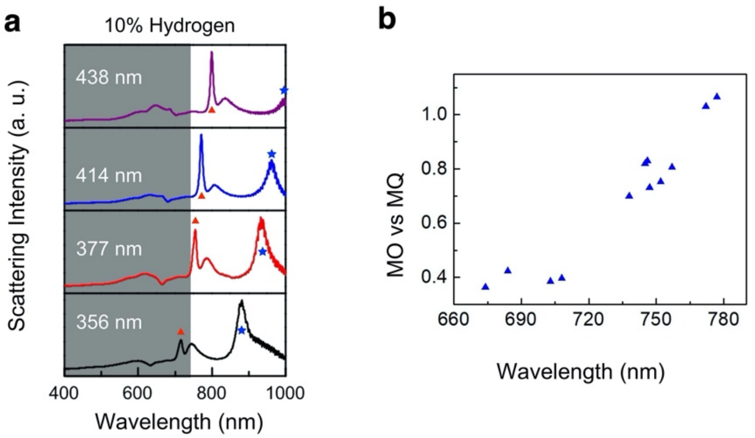

Supplementary Figure 10│ a, Scattering spectra of single a-Si:H(10) NPs. The sizes of NPs are 356 nm (black curve), 377 nm (red curve), 414 nm (blue curve) and 438 nm (purple curve). The red triangles and blue stars label the peak positions of MO and MQ scattering, respectively. **b**, Dependence of MO vs MQ ratio on the peak position of MO scattering for a-Si:H(10).

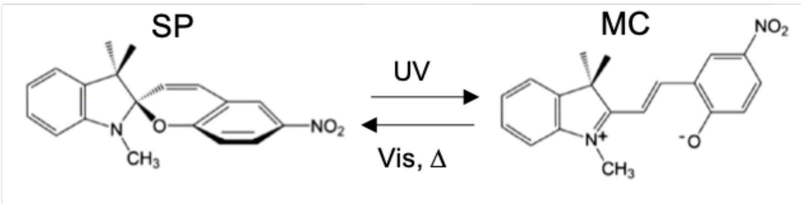

Supplementary Figure 11│ Photochromism of spiropyran. SP and MC represent spiropyran and merocyanine, respectively. UV, Vis and Δ label ultraviolet, visible light and heat, respectively.

Supplementary Note 6. Reversible modulation of the hybrid meta-atom

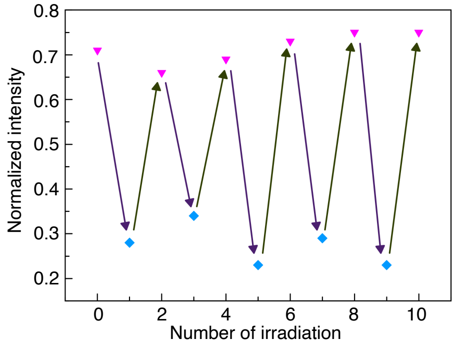

Supplementary Figure 12 | Reversible modulation of the normalized scattering intensity upon alternative UV and green light irradiation. Each irradiation time was 1.5 mins and 3 mins for UV and green light, respectively.

Supplementary References

1. Kuznetsov, A. I., Miroshnichenko, A. E., Fu, Y. H., Zhang, J. & Luk'yanchuk, B. Magnetic light. *Sci. Rep.* **2**, 492 (2012).

2. Fu, Y. H., Kuznetsov, A. I., Miroshnichenko, A. E., Yu, Y. F. & Luk'yanchuk, B. Directional visible light scattering by silicon nanoparticles. *Nat. Commun.* **4**, 1–6 (2013).

3. Lepeshov, S. *et al.* Tunable Resonance Coupling in Single Si Nanoparticle-Monolayer WS_2 Structures. *ACS Appl. Mater. Interfaces* **10**, 16690–16697 (2018).

4. Tribelsky, M. I. & Luk'yanchuk, B. S. Anomalous Light Scattering by Small Particles. *Phys. Rev. Lett.* **97**, 263902 (2006).

5. Kuznetsov, A. I., Miroshnichenko, A. E., Brongersma, M. L., Kivshar, Y. S. & Luk'yanchuk, B. Optically resonant dielectric nanostructures. *Science* **354**, (2016).
